# The Things You Do: Internal Models of Others’ Expected Behaviour Guide Action Observation

**DOI:** 10.1371/journal.pone.0158910

**Published:** 2016-07-19

**Authors:** Kimberley C. Schenke, Natalie A. Wyer, Patric Bach

**Affiliations:** School of Psychology, Plymouth University, Drake Circus, Plymouth, Devon, United Kingdom; University G. d'Annunzio, ITALY

## Abstract

Predictions allow humans to manage uncertainties within social interactions. Here, we investigate how explicit and implicit person models–how different people behave in different situations–shape these predictions. In a novel action identification task, participants judged whether actors interacted with or withdrew from objects. In two experiments, we manipulated, unbeknownst to participants, the two actors action likelihoods across situations, such that one actor typically interacted with one object and withdrew from the other, while the other actor showed the opposite behaviour. In Experiment 2, participants additionally received explicit information about the two individuals that either matched or mismatched their actual behaviours. The data revealed direct but dissociable effects of both kinds of person information on action identification. Implicit action likelihoods affected response times, speeding up the identification of typical relative to atypical actions, irrespective of the explicit knowledge about the individual’s behaviour. Explicit person knowledge, in contrast, affected error rates, causing participants to respond according to expectations instead of observed behaviour, even when they were aware that the explicit information might not be valid. Together, the data show that internal models of others’ behaviour are routinely re-activated during action observation. They provide first evidence of a person-specific social anticipation system, which predicts forthcoming actions from both explicit information and an individuals’ prior behaviour in a situation. These data link action observation to recent models of predictive coding in the non-social domain where similar dissociations between implicit effects on stimulus identification and explicit behavioural wagers have been reported.

## Introduction

Predictions are central to our ability to succeed within an ever-changing environment. They allow us to respond quickly to expected events, to fill in ambiguous or missing information, and to identify mismatches between beliefs and reality, should one’s predictions not come to pass [1–2]. Nowhere are predictions more important than in social interactions, one of the most dynamic situations in everyday life. Predictions help us to coordinate behaviour with others [3], to interpret their actions [4–6] and to detect deception [7]. Indeed, some of the social deficits of autism or schizophrenia may originate from deficits in predicting own and others’ behaviour [8–9].

Prior work has focused on how people derive predictions from social cues and signals, such as emotional expressions [10–11], action kinematics and their match to available tools and goal objects [12–15, 5], object-directed gaze [16], and explicit action goals of others [17–18]. Such cues automatically bias action observation towards the expected actions, allowing rapid and accurate recognition, and planning of one’s own actions relative to the expected future state rather than the current input [19–20, 3]. However, overt signals are not the only source of predictions. Humans are remarkably adept at recognizing other people, with evidence pointing towards dedicated cognitive and neuronal systems for identifying others and storing knowledge about them (e.g., [21–24]). This knowledge not only contains information about their appearance, race and sex, but also information directly related to their behaviour. It has been argued [25–27], for example, that humans form elaborate internal models about the people they know [28–29], describing which behaviours they typically carry out with different objects (e.g., Peter typically goes for chocolate), as well as the mental states these behaviours imply (Peter likes chocolate). Once established, such internal models could be automatically re-activated whenever these individuals are seen again and predict their most likely actions.

It is well established that similar internal models guide our perception of the non-social environment. For example, humans have internalised typical behaviour of objects, such that displacements upward (against the effect of gravity) appear more salient than displacements downward, unless, of course, the object is known to be self-propelled like a rocket [30–31]. Similarly, in natural scenes attention is automatically guided towards the likely locations of relevant objects [32–33] and when identifying items in rapidly presented sequences, internal models predict the forthcoming items [34–35], even when these sequences follow complex second-order rules of an artificial grammar [36]. Together, these findings provide converging evidence that (non-social) perception is not a simple bottom-up process but constantly guided, in a top-down manner, by internal models that specify the behaviour of the external world. On a neuronal level, these influences can be traced to activation in low-level visual areas, which anticipate the incoming stimulation [37]. Behaviourally they manifest in speeded up response times to predictable events, often despite an inability to verbalise the underlying causal structure (for a critical discussion see [38]).

Here, we ask whether a similar mechanism exists for social perception, which makes the current actor’s typical behaviour in the given situation available to guide action observation. Such a mechanism would have to overcome at least two challenges. First, each human act is jointly caused by a number of hidden factors–goals, beliefs, energy and motivation–that observers do not have access to [39]. To an outside observer, others’ behaviour can therefore not be described deterministically but stochastically, in terms of tendencies for action. Second, one of the strongest non-hidden influences on others’ behaviour is the current context, with others’ exhibiting different behaviours in different situations [5, 25, 40–42]. For example, in personality psychology it has been shown that such a situation dependant encoding of traits allows much more robust descriptions of others behaviour than overarching personality traits (i.e., that a child is shy at school but extrovert at home, rather than shy across situations [43]. Person models would need to capture specifically this situation-dependency of human behaviour, encoding the specific intentional behaviour an individual exhibits towards one type of object, but not towards others (e.g., [25]).

Despite these theoretical proposals, there is currently little evidence that action observation recruits such internal person models [28–29]. As noted above, several studies have shown that observers predict others’ actions based on various social cues, such as smiles, gaze, or action kinematics [10–18]. Whilst these cues could indeed exert their effects by providing person information, such as others’ goals and beliefs, they could just as well be explained on the level of action alone, where certain cues (e.g., a smile) directly predict certain behaviours (approach), without drawing upon person information at all (e.g., [44–45]). In contrast, social psychology has shown that people establish person models from behaviour descriptions [46–49]. Yet, while these internal person models have been shown to affect reading times of subsequent behaviour descriptions [50], as well as one’s explicit judgments and memories of these individuals ([51–53], for reviews see [54–57]), their online use during action observation has not been demonstrated. Other studies have shown that people learn others’ looking behaviour towards objects, which then guides attention similarly as directly perceived gaze, and that people automatically activate action knowledge about the people they see [58–59]. However, neither of these studies has demonstrated any predictive impacts on action identification, and the knowledge tested has been very stereotypical, such as the typical behaviours of black and white people [60], the body parts used in sports associated with famous athletes [61–62], or people’s emotional expression when last seen [63]. They therefore fall short of the crucial situation-dependency, which is the hallmark of human action [5, 25, 43].

Here, we develop an experimental paradigm in which such person based predictions can be studied. The studies presented here provide a first test of whether (1) once established, internal models of others behaviour are activated when these individuals are seen again, whether (2) these person models exert a predictive influence on action observation, speeding up the identification of expected actions relative to unexpected ones, and whether (3) they capture the situation-specificity of human action, predicting the actions that others’ typically perform in one situation but not in others. To test these hypotheses, participants were given a simple action identification task, in which they watched the actions of two individuals (John or Claire) in two situations (sitting next to a computer or standing next to a soccer ball). In each situation, they simply reported, with a speeded button press, whether the individual interacted with the object or turned away from it. To induce action expectancies, we either manipulated, unbeknownst to participants, the actual frequencies of the two individuals’ behaviours across situations in Experiment 1 (e.g., Claire would be more likely to kick a soccer ball than type on a computer and vice versa for John), or we gave them explicit descriptions (“gossip”) of how the two individuals would behave in Experiment 2.

This paradigm captures both the required stochastic rather than deterministic distributions of others’ actions, and their dependency on situational context (i.e., it is not the case that one person simply interacts more than the other, but rather that each person has a specific interaction “signature” across objects). It allows us to test whether internal models of the two individual’s behaviour are automatically activated when we watch other people and predict their most likely forthcoming action in the given situation. Even though task irrelevant, the identity of the current actor–and the way in which we have previously observed them behaving with the objects–should then directly affect action observation. Actions should be identified more quickly and accurately if they are typically carried out by this individual in the given situation, compared to actions that are overall equally frequent but are typically carried out by someone else. This is exactly what we find in both experiments.

## Experiment 1: Predictions Derived from an Individual’s Prior Behaviour

Experiment 1 provides an initial test of whether, once established, internal models of other individual’s typical behaviour are automatically activated whenever they are seen again and facilitate identification of their most likely forthcoming actions in the given situation. Participants performed a simple action identification task, in which they reported, with speeded button presses, whether an actor interacted with or turned away from an object, while both the situational context (in front of a computer or a soccer ball) and the identity of the actor (Claire, John) varied. Unbeknownst to the participants, the two actors had different behaviour profiles such that they were differentially likely to interact with each object (e.g., Claire would be more likely to interact with a soccer ball than with a computer and vice versa for John) whilst the overall action frequency was controlled. If observers establish internal models of the two actors’ typical interaction signatures across situations and activate them whenever they are seen again then actor identity should directly affect action observation: actions should be identified more quickly and accurately when carried out by an individual that typically performs this action in the given situation, compared to an individual that carries it out more rarely, even though both actor and object were task irrelevant.

To measure the extent to which such effects depend on explicit knowledge or response strategies of participants, we asked all participants in a funnel debrief whether they noticed any patterns in the stimuli. In addition, we asked them to rate which objects they thought the two individuals “liked” to interact with more. These two questions provide potentially dissociable information [38]. Question 1, whether participants had detected the manipulation, tests for spontaneous awareness of the manipulation during the experiment which participants could have relied on to guide strategic responses. In contrast, the liking question tests for whether any tacit information about the two individuals’ behavioural tendencies can be explicitly accessed, in principle, when participants’ now-formed person models are appropriately probed. Such responses typically do not reflect explicit knowledge about the global co-variation patterns, but the generation of such knowledge at the time of probing, perhaps by bringing to mind remembered instances of the seen stimuli [38]. In other words, while participants might not have independently detected the contingency patterns during the experiment (Question 1), they might be able to make accurate judgments by “reading out” the acquired internal models retrospectively (Question 2).

We tested these hypotheses in a first group of participants (Experiment 1a) and then replicated in a second, near-identical study (Experiment 1b), which only differed in whether participants were asked to rate, as in Experiment 1a, which object they perceived the two individuals to like more (subgroup 1 of Experiment 1b), or whether they were asked to rate which object they did, in fact, interact with more (subgroup 2 of Experiment 1b).

## Method

### Participants

Forty-two undergraduates from Plymouth University (31 females, 37 right-handed, mean age = 20.40 years, SD = 3.71 years) took part in Experiment 1a and fifty-seven in Experiment 1b (49 females, 51 right-handed, mean age = 20.39, SD = 5.56 years), in exchange for course credit. In both experiments, participants were excluded from response time and error rate analysis if they detected the experimental manipulation (Exp. 1a, n = 3, Exp. 1b, n = 2), or if they made more than 10% errors (Exp. 1a, n = 2). Sample sizes were determined with G-Power [64] on pilot data from different participants (*n* = 42), which indicated that a sample size of at least 36 was required to reliably detect a main effect of Expectancy (dz = .625) with .95 power. All experiments were approved by the Faculty of Health and Human Sciences Research Ethics Committee prior to data collection, and we report all measures, manipulations and exclusions for all experiments. All participants provided written consent.

### Materials and apparatus

The autism quotient scale (AQ; [65]) contained 50 questions to measure the presence of autism-like traits in neurotypical individuals. No relationships were found between autism-like traits and any of the effects in the experiments and will not be discussed further.

**Fig 1:**
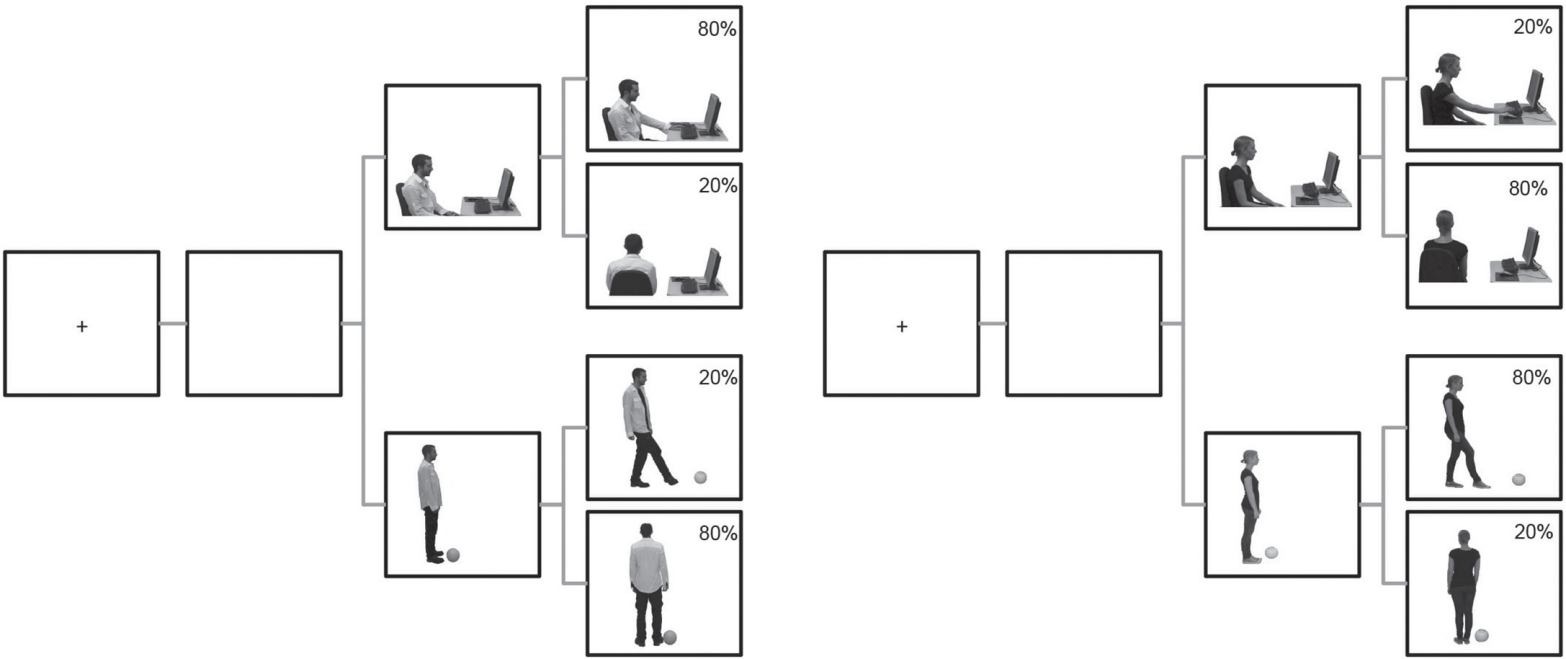
Trial sequence. Each trial started with a fixation cross (400 ms.) and a brief blank screen. Each action started with an image showing one of the two individuals (John, left; Claire, right) in one of the two situations (at a computer, top; near a soccer ball, bottom). They then either interacted with the object or turned away from it, with one individual typically interacting with one object and turning away from the other, and vice versa for the other individual.

The experiment proper was controlled by Presentation (Neurobehavioral systems, Inc; version 14.9, Build 07.19.11) using a Windows XP SP3 1280x1024 32 bit colour 17” display. The stimulus set consisted of 16 different two frame sequences. Each sequence consisted of a neutral image, which showed one of the two actors (John, Claire) in one of the two situations (next to a computer, next to a soccer ball), displayed for 500ms. This image was identical for both actions that might follow (interact, turn away) and served as a prime for the identity of the individual. The second image then showed the actor either interacting with this object (typing on the computer, kicking the soccer ball) or turning away from it. The two images were presented without an inter-stimulus interval, creating the impression of apparent motion [66]. Static images rather than video clips were used to remove, via photo-editing, all cues for context so that only the object and actor were influential. This also provided unambiguous onset times for the action judgments (the second image in the sequence). To control for Simon-like [67] response effects, in one half of the trials, the object was to the left of the individuals, and in the other half on the right (see Fig 1 for an example of the stimuli).

In the first exit questionnaire, participants in Experiment 1a and one half of participants in Experiment 1b rated how much each actor liked each object on a scale from -4 to 4 with no zero point e.g., “How much do you think John liked the ball?”. The second half of participants in Experiment 1b rated how much each actor interacted with each object using the same scale (-4 to +4 with no zero point). The second exit questionnaire was a funnel debrief consisting of five questions identifying any explicit knowledge of the experimental manipulation that could guide strategic responses. They were first asked “How easy did you find the task of identifying whether the actors interacted or turned away from the object?” and answered this by circling a number between 1 “really difficult” and 10 “really easy”. They were then asked: “Did you find one actor easier to identify than the other? If so please state which one.”, “Did you find one action easier to identify than the other? If so please state which one.”, “Did you notice anything unusual about any of the actors or objects?”, and “Did you notice any patterns in the stimuli?”

### Procedure

Participants completed the AQ and then received written and verbal instructions. When the experimenter was satisfied that the task was understood, participants completed the computer task, which contained 240 trials. Both actors (John, Claire) were shown equally often in each of the situations (computer, soccer ball), but we varied how often they performed the two possible actions in these situations (interacting, turning away). In 80% of the trials, the actors would perform their typical action while in the remaining 20% they would perform the atypical action. Thus, for one participant, John would interact with the computer in 80% of the cases and turn away from it in 20%, while he would turn away from the soccer ball in 80% of cases and interact with it in 20%. Claire would show the reverse contingences (interact with the soccer ball in 80% and the computer in 20% of cases). These contingency mappings were counterbalanced across participants. The trials were presented in blocks of 40 (four repetitions of the eight regular trials and one set of the oddball trials) to ensure an equal distribution of oddballs across the experiment.

Each trial started with a fixation cross in the centre of the screen (400 ms). After a blank screen of 400 to 800 ms (randomly chosen), one of the two frame sequences was presented. In Experiment 1a, the stimulus onset asynchrony (SOAs) between the first and the second frame of the action sequences was either 150 ms or 850 ms. Because no effects depended on SOA, in Experiment 1b, the images followed each other with a fixed SOA of 500 ms. Participants pressed the “UP” arrow key to identify that the actors were interacting with the objects (either typing or kicking) and the “DOWN” arrow key to identify that the actors were turning away from the objects. Participants were asked to respond as quickly and as accurately as possible. If they took longer than 2000ms or responded incorrectly, an error message reminded them of the correct button assignment. After the experiment, participants completed the two exit questionnaires, were thanked and fully debriefed.

### Trial exclusions

The same exclusion criteria were used across all experiments. The first twelve trials of each experiment were considered training trials and excluded. Additionally, trials were excluded if they fell within any of the below criteria: 1) trials with RTs greater than 2000ms (maximum duration of the response interval), 2) trials with anticipations (i.e., responses before the critical second frame was displayed), 3) trials where Presentation timing was uncertain (measurement uncertainties larger than 10 ms), and 4) trials with RTs over 3 standard deviations from this participants’ condition mean. For the analysis of RTs, error trials were additionally excluded.

## Results

4.58% of trials were excluded in Experiment 1a and 1.28% from Experiment 1b (see above for criteria). The remaining data were analysed with a repeated measures ANOVA with the factors Observed Action (act with object, turn away from object) and Action Typicality (typical, oddball), separately for response times (RTs) and error rates.

### Response times

**Fig 2:**
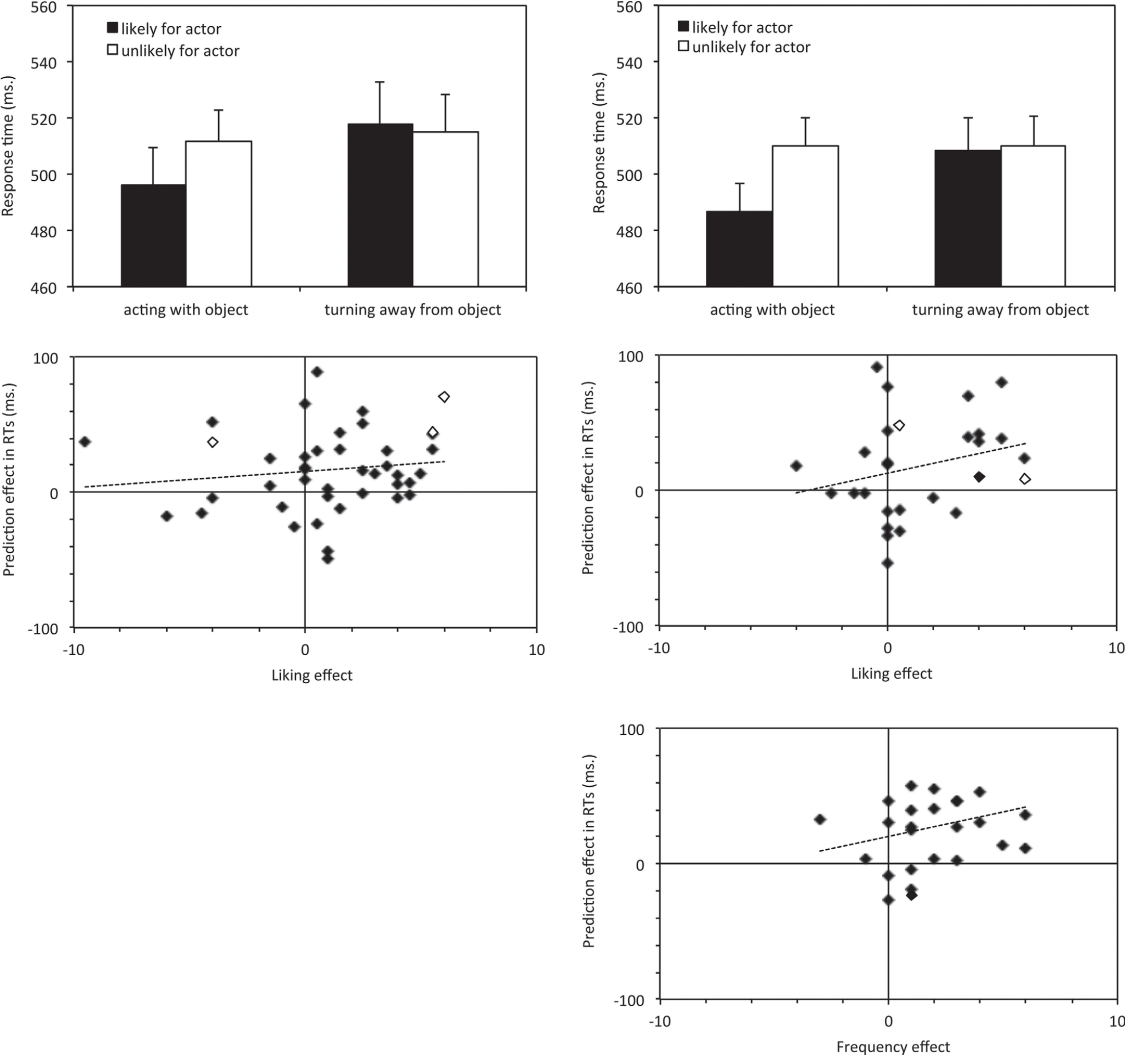
Experiments 1a and 1b RT and liking results. Top panel: average response times in Experiment 1a (left panels) and 1b (right panels). In each panel, the left bars show identification of actions towards objects (typing on a computer or kicking the soccer ball) and the right bar shows withdrawals from these objects. The black bars reflect actions expected of this individual in the given situation, and white bars show the action expected of the other individual. Error bars show the standard error of the mean. Middle and bottom panels: correlation between prediction effects in the RTs for actions towards objects and the corresponding differences in perceived object liking and interaction frequency, for individuals who either identified (unfilled diamonds) or did not identify (filled diamonds) the behavioural pattern.

The analysis of Experiment 1a revealed no main effect of Observed Action, *F*[1,36] = 2.233, *p* = .144, *ηρ*^*2*^ = .058, but a marginally significant main effect of Action Typicality, *F*[1,36] = 3.140, *p* = .085, *ηρ*^*2*^ = .080, as well as an interaction between both factors, *F*[1,36] = 6.378, *p* = .016, *ηρ*^*2*^ = .151. As can be seen in Fig 2, actions towards objects (kicking a soccer ball, typing on the computer) were identified more quickly when the current actor typically carried out these actions with the objects, compared to when they were atypical for the actor, *t*[36] = 3.330, *p* = .002, Cohen’s *d* = .16. However, no such effect was found for withdrawals, *t*[36] = .518, *p* = .607, *d* = .04.

The analysis of Experiment 1b fully replicated these findings. It revealed a marginally significant main effect of Observed Action, *F*[1,51] = 3.070, *p* = .086, *ηρ*^*2*^ = .057 and the predicted effect of Action Typicality, *F*[1,51] = 12.314, *p* = .001, *ηρ*^*2*^ = .194. Importantly, as in Experiment 1a, this effect was qualified by an interaction of both factors, *F*[1,51] = 12.773, *p* = .001, *ηρ*^*2*^ = .200. The RT advantage for typical relative to atypical actions was only present when the individuals acted with the objects (kicking a soccer ball, typing at a computer), *t*[51] = 4.620, *p* < .001, *d* = .29, but not when they withdrew from them, *t*[51] = .379, *p* = .707, *d* = .021. Entering Group (liking questions, frequency questions) into the ANOVA did not reveal any further effects, all *F* < 1.

### Error rates

No effects were found in either Experiment (*F*s < 2.124) for the error data, with the exception of a main effect of Observed Action in Experiment 1b, *F*[1, 49] = 5.155, *p* = .028, ηρ^*2*^ = .095, with more errors for actions towards objects then withdrawals, which was unrelated to our hypotheses.

### Anticipations

An important question is whether internal person models only affect action identification times, or whether it also causes overt response anticipations, such that participants identify the expected action even though it is not yet presented (i.e., while the neutral image is still on the screen). Due to the low number of anticipations (6.46% in both experiments), we combined the data for Experiments 1a and 1b to increase power and performed a repeated measures ANOVA with the factors Expected Action (act with object, turn away from object) and Response (typical action identified, atypical action identified) on the data from the participants who made at least one anticipation, *n* = 31. There was a marginally significant main effect of Response, *F*[1,30] = 3.214, *p* = .083, *ηρ*^*2*^ = .097, revealing that responses typically anticipate the expected action, but no main effect of Expected Action (*F* = 2.161), nor an interaction between the two (*F* = .171). Thus, the anticipations show that others’ typical behaviour does not only affect action identification, but also sometimes causes participants to anticipate the forthcoming response while the neutral image was still on the screen.

### Liking and frequency ratings

After completing the action identification task, participants rated how much the two individuals liked the two objects (in Experiment 1a, and subgroup 1 of Experiment 1b), and how much each individual had interacted with them (subgroup 2 of Experiment 1b). In Experiment 1a, objects that were typically acted upon by the given individual were rated as more liked by this individual (*M* = 1.79, *SD* = 1.52) than objects this individual typically turned away from (*M* = -0.17, *SD* = 1.89), *t*[36] = 3.818, *p =* .001, *d =* 1.05. This effect was replicated in Experiment 1b. When the objects were typically acted upon they were rated as more liked (*M* = 1.74, *SD* = 1.21) than when they were turned away from (*M* = 0.40, *SD* = 1.82), *t*[26] = 2.498, *p =* .019, *d =* .80. Similarly, when the objects were typically acted upon they were rated as being interacted with more (*M* = 2.20, *SD* = 1.06) than when they were turned away from (*M* = 0.50, *SD* = 1.49), *t*[24] = 4.332, *p* < .001, *d =* 1.30,

Having established that participants can access some explicit information about the two individual’s behaviour, we then tested whether explicit person knowledge predicts the effects during action identification. We therefore also included those participants that explicitly detected the contingencies between individuals, objects and actions (but also report if results depend on these participants). A regression analysis measured the relationship between apparent explicit awareness of individuals’ behaviours as seen in the liking and frequency ratings, and the response time effect for actions towards objects (difference between likely and unlikely actions for the actor), for each of the three participants groups separately. None of the three participant groups showed a significant correlation, (Exp 1a, *r* = .187, *n* = 40, *p* = .248; Exp 1b liking, *r* = .252, *n* = 28, *p* = .195; Exp 1b, frequency, *r* = .210, *n* = 26, *p* = .304). However, in each, the intercept was different from zero (Exp 1a, *t* = 3.325, *p =* .002; Exp 1b liking, *t* = 1.710, *p =* .099; Exp 1b, frequency, *t* = 2.117, *p =* .045), indicating that even those with no apparent explicit awareness in the liking or frequency ratings still showed significant RT prediction effects. The same pattern is seen when three participants who detected the manipulation were excluded, with the exception that the intercept for the liking ratings in Experiment 1b now failed to reach marginal significance (*t =* 1.519, *p* = .142).

To attain enough power to detect weaker correlation effects, the data from all three subgroups were pooled. These analyses indeed revealed a marginally significant correlation between the post-experiment ratings and the response time effects (all participants, correlation; *r* = .186, *p* = .073; unaware participants only, correlation; *r* = .181, *p* = .089). In addition, they confirmed the significant intercept (all participants, *t =* 4.957, *p <* .001, unaware participants only, *t =* 4.544, *p <* .001), indicating that even those who were unable to explicitly recall the individuals’ behaviour still showed reliable response time prediction effects.

## Discussion

Experiment 1 tested whether internal models of others’ typical behaviour are automatically re-activated whenever they are seen again, and predict their most likely forthcoming actions in the given situation. Indeed, actions were identified more rapidly when they were typical for the given individual in the given situation, compared to an action that is, overall, equally frequent but typically carried out by another individual. These effects of actor identity on action observation were found even though individual and situation were task irrelevant, and the overall frequency of each action was controlled across situations and individuals. As such, they provide first evidence that watching other people goes along with activation of internal person models that describe these individual’s typical behaviour in the given situation, which biases identification towards their most likely forthcoming action.

A striking observation was that in both experiments action expectations affected the identification of actions with objects (e.g., kicking the soccer ball, typing on the keyboard), but not withdrawals from them. Although not predicted, this finding is in line with the proposal that action prediction specifically occurs for meaningful actions towards objects, ([for a recent review, see [5]; see also [13–14, 25]), and that object avoidance is coded on a second-level, as an inhibition of a potential approach [68–69]. For example, even though there are neuronal populations for representing intransitive action [70–71], the majority of mirror neurons, one of the proposed core mechanisms of action understanding and prediction, fire only for actions towards objects (for a review see [72] and even in humans object-directed actions are represented in dedicated neuronal populations [73]). Indeed, studies in humans show that afforded interactions with an object are perceived and predicted more readily than non-afforded actions [19, 74] and studies on high level mentalizing abilities show that predictions of what other people will do (e.g., in theory of mind tasks) occur for approach related behaviours but not for avoidance behaviours [68–69]. Our results are therefore in line with these studies and further support the special status of object-directed actions in action observation and prediction.

The effects of actor identity on action identification are unlikely to result from strategic responses of participants that detected the experimental manipulations. Only five participants spontaneously detected the experimental manipulation when probed after the experiment. Moreover, if the effects reflected strategic response preparation they should have been found not only for interactions with objects, but also for withdrawals, especially as overall response times between these conditions did not differ. Yet, faster responses were only found for meaningful actions towards objects, but not actions away from them. Finally, explicit response preparation effects should specifically affect error rates, not only response times [75–76], but no such effects were detected.

Importantly, though, when explicitly probed after the experiment, participants could make reliable judgments about which objects the two individuals liked more and which they tended to interact with. Thus, if John was typically seen interacting with the computer but turning away from the soccer ball, participants were able to retrieve this information when directly prompted, and he was later judged to like computers more than soccer balls. Importantly though, while the data reveal some weak relationships between these post-experiment ratings and prediction effects in response times, they also showed that even those participants that did not show any rating effect still showed significant prediction effects. This finding is in line with the idea that explicit knowledge is not the basis for the prediction effects but that, instead, these internal models of others’ behaviour are not fully opaque, but can be accessed to generate behaviour information, perhaps by playing through relevant instances in memory (for similar dissociations, see [77–79]).

## Experiment 2: Explicit Knowledge of Others’ Behaviour

Action observation is not our only source of information about our interaction partners. People love to gossip (e.g., [80]) and mutual acquaintances are a rich source of information about other people, which might exert similar predictive influences on action observation. This is also the typical situation tested in prior work in social psychology where the influence of explicit person descriptions on subsequent person memory and reading times were tested [81, 51, 53, 56–57]. The current study attempted to capture this explicit social knowledge, and tested whether such explicitly derived person models have similar or different effects on action observation as the actual behaviour pattern of the individuals in Experiment 1, and whether they interact with this (potentially conflicting) information. At the start of the experiment, participants were given an explicit description about the two actors’ typical behaviour (“George typically kicks the ball but rarely types on the computer”). They then performed the same action identification task as in Experiment 1. Across blocks within the experiment, the actual behaviour tendencies could either follow the person description (in 75% of the trials the individual acts according to expectations and counters the expectations in the remaining 25% of trials), conflict with the prior description (the individuals’ actions counter the expectation in 75% of the trials), or could be equivocal (they carried out the described action in 50% of the trials and the alternative action in the other 50%). To ensure that participants would perceive the individuals’ behaviour in light of the prior behaviour descriptions, we asked them to assess, after each block, to what extent individuals’ actual behaviour matched the initial description.

This task therefore pits implicitly derived internal models of other people from those derived by explicit information. It allows us to test, first, whether explicit information about others leads to similar biases in identifying their actions as found for implicit statistical manipulation of their behaviour. Second, it allows us to test the extent to which explicit and implicit predictions interact or are independent of one another.

## Method

### Participants

49 participants (39 females, mean age = 20.92 years, SD = 6.06; 44 right-handed) took part in the study in exchange for course credit. One participant was excluded for making more than 10% errors.

### Apparatus & Materials

Stimuli and the course of each trial were identical to the previous experiments. The experiment was controlled with E-Prime 2.0 (Psychology Software Tools, Pittsburgh, PA), and responses were recorded with button boxes.

### Design and Procedure

Participants received detailed instructions then underwent 16 explicit practice trials in the action identification task of Experiment 1b (more practice trials were needed here than in the previous experiment due to the increased difficulty highlighted by pilot testing). All actions in the practice trials were carried out by a third actor who did not appear in the main experiment. Participants were then informed that whilst performing this task they would also be asked to perform a second task and assess whether a person description matches the individual’s actual behaviour. For practice, they were informed that the actor typically kicked the ball but turned away from the computer. They then underwent 12 further practice action identification trials, in which 8 of the trials supported the hypothesis and 4 contradicted it. They then rated how much they agreed or disagreed that the seen behaviour corresponded to the prior person description on a 4-point scale (1 = “completely disagree”, 4 = “completely agree”).

After both participant and experimenter was satisfied that the task was understood, the participant was given an explicit description about the actors’ typical behaviour (e.g., that John typically kicks the ball but turns away from the computer, or vice versa, and that Claire has the opposite preferences), and that they had to evaluate the appropriateness of this behaviour description after seeing the individuals’ actual behaviour in each of the experiments’ nine blocks (32 trials each). At the start of each block participants were reminded of the explicit person description and that this was a new set of trials and to ignore what they had seen previously. They then performed the action identification task of Experiment 1b. The individuals’ actual behaviour differed in each block, such that it could either conform to the prior person description (75:25), be equivocal (50:50), or contradict it (25:75), such that the actors performed the opposite action more frequently. At the end of each block, participants rated whether they agreed that the individuals’ behaviour corresponded to the person descriptions at the start of the experiment. Block order was randomised across participants.

At the end of the experiment, the social intelligence scale [82] was administered. The scale consists of 21 questions each on a 7 point Likert scale. Examples of questions are “I can predict other peoples’ behaviour”, “I often feel uncertain around new people who I don’t know” and “I can often understand what others mean through their expression, body language, etc.” There were no significant correlations between this scale and the effects seen and so this will not be discussed further.

## Results

### Response times

7.26% of trials were excluded in total (5.33% errors and 1.93% for RTs greater than 3 SD from the mean). The remaining data were analysed with a repeated measurements ANOVA with the factors Action-Description Match (the observed action follows/does not follow the person description), Block-Description Match (observed statistics in the current block matches the person description, are equivocal, contradict the description), and Action Type (act toward object, withdraw from object).

**Fig 3:**
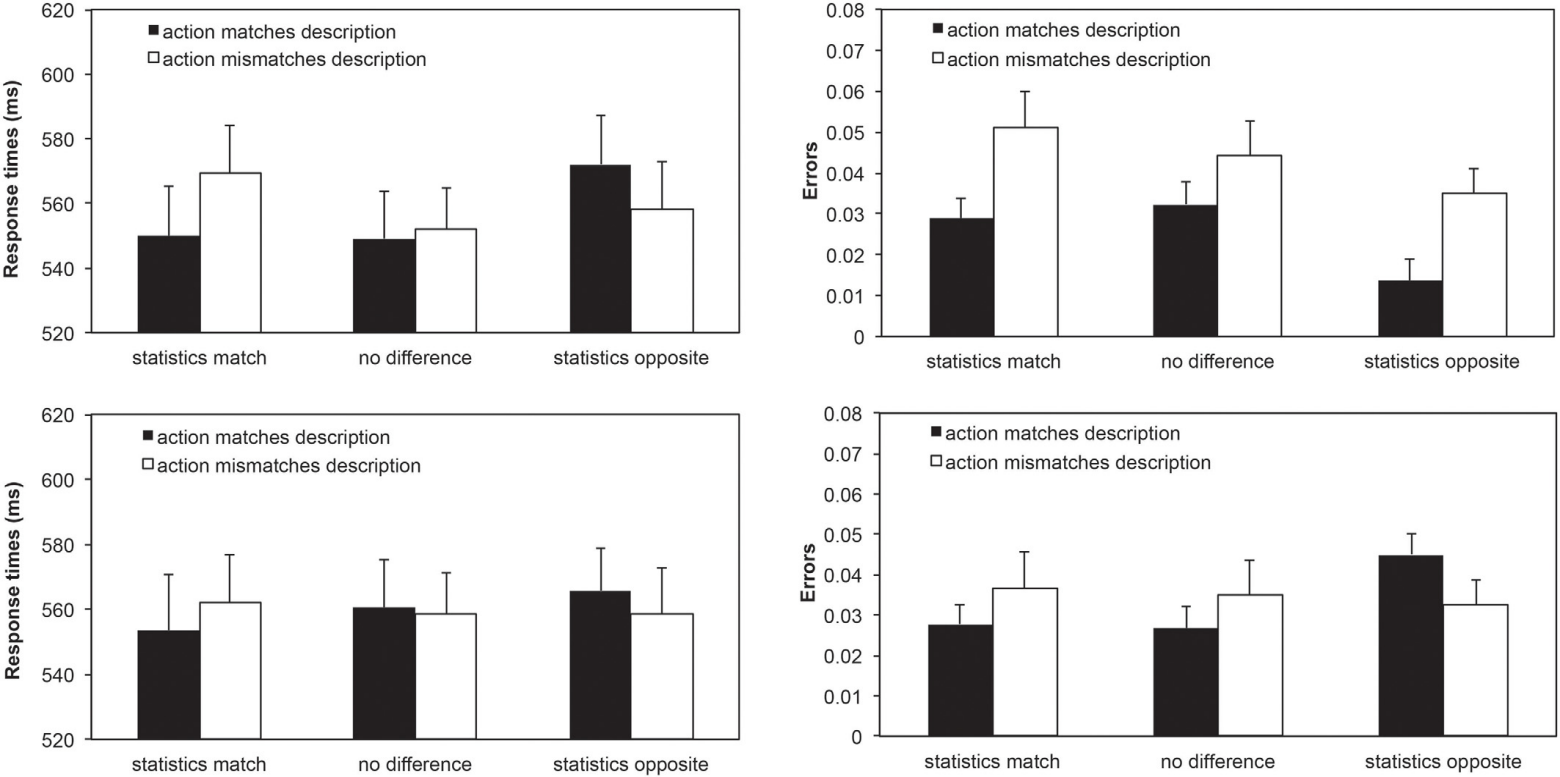
RTs and error rates for Experiment 2. The black bars represent trials which followed the hypothesis and the white bars represent trials, which are the opposite of the hypothesis. The left side shows the response times and the right side shows error rates. The top row indicates actions towards objects and the bottom row indicates withdrawals. Error bars show the standard error of the mean.

The analysis of RTs (Fig 3, left panels) revealed no main effect of Block-Description Match, *F*[2,44] < 1, nor of Action-Description Match, *F*[1,47] < 1, nor of Action Type, *F*[1,47] < 1, providing no evidence that actions that matched the explicit information were generally identified more quickly than mismatching actions. Importantly, there was an interaction between Block-Hypothesis Match and Action-Hypothesis Match, *F*[2,46] = 5.062, *p* = .010, *ηρ*^*2*^ = .180, signalling that response times were driven by the statistical regularities in a block, but not the explicit person description. In a block in which the individuals’ behaviour matched the prior description, participants more quickly identified actions that matched this description (*t* = 2.260, *p* = .029, *d* = .66). However, in blocks where the action likelihoods were equal (and the individuals acted randomly), there was no differences between actions that matched or mismatched the prior person description (*t* < 1). Finally, when the actors’ behaviours in a block contradicted the person description, the effect reversed, too, with RTs being faster for trials that mismatched the person description (but therefore matched the statistics in the block), *t* = 2.060, *p* = .045, *d* = .60. These data therefore reveal no effect of prior explicit person information but replicate Experiment 1 and show that internal person models derived from an individual’s action likelihoods affect response times even within relatively short blocks of 32 trials. There were no other effects (*F*s < 2.391). In Experiment 1, action likelihood specifically affected actions with objects but not withdrawals from them. We therefore tested whether the RT effects are similarly driven by these actions towards objects. Indeed, planned comparisons revealed no significant effects (all *F*s<1.376) for withdrawals, while the acting towards trials showed the relevant interaction between Block and Hypothesis Match, *F*[2,46] = 4.471, *p* = .017, *ηρ*^*2*^ = .163.

### Error rates

Error rates were analysed with the same ANOVA model as the RTs. It revealed no effect of Block-Description Match, *F*[2,46] = 1.099, *p* = .342, *ηρ*^*2*^ = .046, nor of Action Type, *F*[1,47] = .360, *p* = .552, *ηρ*^*2*^ = .008, but a main effect of Action-Description Match, *F*[1,47] = 7.404, *p* = .009, *ηρ*^*2*^ = .136. Participants made more errors when actions conflicted with the prior person description. This effect was qualified by an interaction of Action-Description Match and Action Type, *F*[1,47] = 6.385, *p* = .015, *ηρ*^*2*^ = .120, showing that the increase of errors for non-expected actions was stronger for actions towards objects than withdrawals, as was found in the previous experiments for statistical person information. Indeed, pairwise comparisons showed that the main effect of Action-Description Match was present for actions towards objects, *F*[1,47] = 15.740, *p* < .001, *ηρ*^*2*^ = .251), but not for withdrawals (*F* < 1.265). Finally, there was an interaction of Block-Description Match and Action Type, *F*[1,47] = 3.790, *p* = .030, *ηρ*^*2*^ = .012 but this was not relevant to our hypotheses.

### Behaviour ratings

Data to what extent the individuals were rated to have followed the person description in the different blocks were analysed with a one-way ANOVA with the factor Block-Description Match (blocks either matched the hypothesis, mismatched the hypothesis or showed each action equally). This main effect was significant, *F*[1, 143] = 72.053, *p* < .001, showing that participants reliably distinguished the different behaviour patterns in the three types of blocks. Agreement was higher when the actors’ behaviour in a block matched the prior person description (*M =* 2.97, *SD =* .51) than when action likelihoods were equivocal (*M =* 2.27, *SD =* .36), *t*[47] = 8.201, *p* < .001, or opposite to the description (*M =* 1.93, *SD =* .42), *t*[47] = 10.391, *p* < .001. Moreover, they were higher for equivocal likelihoods then distributions opposite to the person description, *t*[47] = 6.096, *p* < .001.

## Discussion

Experiment 2 showed that internal models of others’ behaviour can be established either from observing their typical behaviour or from explicit person descriptions, with both having dissociable effects on action identification. The two individuals’ action likelihoods affected action identification times (but not error rates), such that actions towards objects were identified more quickly when they were typically carried out by this individual in the given situation, compared to actions that were carried out more rarely. In addition to replicating Experiment 1, these findings show that person models can be established from relatively few exposures (32 trials in a block) and exert their influence spontaneously, despite the secondary task of assessing the individual’s behaviour.

In contrast, explicit behaviour descriptions about the two individuals directly affected error rates (but not response times), such that an action was more likely to be misidentified for the action that was currently expected (e.g., identifying a kick as a withdrawal when a withdrawal was expected). This effect on error rates is striking given that the participants were aware that across blocks the actions were equally likely to match and mismatch the behaviour description, and that they were instructed to evaluate whether the explicit information was correct or not. Merely maintaining a hypothesis about someone else’s behaviour may therefore induce a subtle tendency to act according to this prediction, even when explicitly trying to keep an open mind.

The differential effect of explicit and implicit information is consistent with other research on predictive coding in the non-social domain. Implicit information about statistical regularities often affects response speed, perhaps because it allows relatively low-level perceptual (or motoric) anticipations of forthcoming events [76]. Explicit information, in contrast, provides higher-level assumptions that might induce a tendency to make explicit “wagers” about what will be observed, which allows people to overtly test any hypothesis they have about the regularities guiding the events against reality [83, 75]. Indeed, in a recent study, it was exactly these explicit behavioural wagers that were associated with the explicit (rather than implicit) recognition of the underlying rules that governed the event sequences [76]. As such, the present dissociations are in line with predictive coding work that sees behavioural wagers as key learning mechanisms for explicit learning and verification of explicit hypotheses. In addition, it further confirms that the response time effects do not reflect this currently available (and explicitly tested) person model, but rather statistical information about other’s most likely behaviour with the different objects.

## General Discussion

In two experiments, we tested whether observers use internal models about other individuals’ typical behaviour in different situations to predict their most likely forthcoming actions. In Experiment 1, participants performed a simple action identification task–whether the actor interacted or withdrew from an object–while we manipulated, unbeknownst to them, the probability distribution with which the actors performed these behaviours across situations (i.e., one individual typically interacted with a soccer ball but withdrew from a computer, and vice versa for the other individual). We found that action identification was indeed sensitive to actor identity, being faster for actions that were typical for an individual in a given situation, compared to actions that were overall equally frequent but typically carried out by someone else. This effect was found even for participants that were unable to report the individuals’ typical behaviours and even though both situation and person were task irrelevant, suggesting a largely automatic effect.

Experiment 2 then showed that similar–but dissociable–effects are evoked for explicit information about the acting individuals. Here, participants evaluated behaviour descriptions about the actors while we varied, in different blocks, the extent to which the actors indeed followed these patterns. We found that the actor’s actual action likelihoods again sped up identification times, showing that these prediction effects adjust to new statistics within very few exposures (32 trials within a block). In contrast, explicit behaviour descriptions affected error rates, causing participants to respond according to the explicit predictions instead of what was perceived. This bias was observed even though participants were asked to merely evaluate the given behaviour descriptions, and were aware that the actual behaviour may differ. Simply evaluating a hypothesis about others’ behaviour may therefore induce an involuntary confirmation bias [84] towards these actions irrespective of the actual behaviour patterns, or involuntary behavioural “wagers” where participants test their explicit action hypotheses against reality ([76], see also [83, 75]).

The two experiments provide converging evidence that internal person models influence action observation in a predictive manner, such that the actions others are most likely to carry out are identified faster and more accurately. These findings are in line with recent theoretical work that has re-conceptualised social perception, away from conventional bottom-up mechanisms that match kinematic information to own action knowledge [85–87] towards interactive models, in which action observation is constantly guided by prior knowledge [4–6]. As found here, these models assume that top-down information about the person (action tendencies, goals, beliefs) is constantly integrated with the situational constraints (objects available for goal achievement) to predict the most likely actions. Expected actions should therefore be processed rapidly, while unexpected actions cause salient prediction errors and revisions of one’s person models. While previous research has revealed that such expectancies are derived from social cues (e.g., [10, 13–16, 19, 88]), the present data reveal an influence of actor identity and the internal models of their behaviour: what we were told about them and how they have responded in the same situation before.

To our knowledge, this is the first study to show such a top-down effect of person models on online action observation. While it is known that person models provide a reference frame against which others’ behaviour can be judged [46–48], prior studies used measures that were far removed from online action observation, such as reading times or memory about individuals [51, 53, 55–57, 81]. Other studies have shown that people re-activate action-related information about others whenever they are seen, but this knowledge has been very general and does not reflect how these individuals behave in different situations (e.g., the body parts used in the sport of famous athletes, [61–62]; prior emotional expression or direction of gaze, [63, 58–59]). Our new data now show, first, that internal person models can affect online action identification, and that, second, this person knowledge is organised around discrete situations, reflecting not only *what* somebody typically does, but also in *which situations* these actions occur [25].

A striking finding was that the effects on online action identification were largely de-coupled from the participants’ ability to make explicit judgments about the two individuals’ behaviour. In Experiment 1, participants were able to accurately judge how frequently the individuals had interacted with the objects and how much they “liked” these activities. Yet, while these judgments were weakly related to the response time effects, the speed up for expected actions was found even in those who were unable to make such judgments. Similarly, in Experiment 2, while giving participants explicit knowledge about the individuals also led to overt biases in action identification, it did so differently than implicitly acquired person knowledge, affecting error rates instead of response times, causing participants to sometimes respond in line with their expectations rather than observed reality.

Similar dissociations are also known from social psychology. The explicit judgments that people make about others are often abstracted away from the behaviours that were actually observed, leading to a similar lack of strong correlational relationships as observed here [77–78]. It has therefore been argued that participants might not make explicit judgments during social perception at all. Only when explicitly asked after the experiment, they form such impressions in an ad-hoc manner, by relying on their (imperfect) memory of what was previously observed. In such a view, the response time effects in Experiment 1 reflect the automatic generation and activation of person models when these individuals are seen. The post-experiment explicit rating effects, in contrast, reflect attempts to “read out” these models, in a retroactive fashion, perhaps by simulating/imagining the observed events that one has previously observed, and drawing conclusions about them.

This interpretation is also in line with research on causal or statistical learning. People are able to learn even complex second-order relationships between events, and respond faster to expected stimuli, compared to unpredicted ones. In many cases, this knowledge cannot be explicitly verbalised by participants and even if they can, it is not diagnostic of prediction effects in the response time task (e.g., [89–90]; for a critical view, see [38]). As argued above, this does not mean that there are two separate systems for implicit and explicit learning. Instead, it might suggest that participants solve explicit tasks by trying to re-activate their internal models based on the cues provided, but that this re-activation is imperfect and differently effective in different individuals (e.g., [89, 91–92, 38]).

The observed distinction between implicit and explicit knowledge affecting response times and error rates, respectively, is particularly in line with such models. Recent work in predictive coding suggests that implicit information about statistical regularities often affects response speed, because it might support relatively low-level perceptual (or motoric) anticipations of forthcoming events [76]. In contrast, explicit hypotheses about forthcoming events provide higher-level inferences that were constantly tested with explicit behavioural “wagers”, which are then either confirmed or disconfirmed through the errors participants make ([76]; see also [75, 83]). Our results therefore suggest that social expectations may be similarly tested against the individual’s actual behaviour.

Whilst social research demonstrates the continued influence of initial trait expectancies on categorising behavioural descriptions (e.g., [48. 53, 57]), in Experiment 2 we find that observed actions very quickly adjust the initial hypothesis, such that participants are able to judge the actual behaviours accurately after each block. This may be because the more concrete actions of our research have a stronger “updating” effect, or that the described behaviours (“John will mostly type on the computer, but turn away from the soccer ball.”) are more specific than the trait expectancies of the social literature which tend to be much more general and thus more robust against incongruent behaviours (for reviews see [54–57]). However, it could also be that most social studies generally do not investigate effects while impressions are still being formed. Indeed, one study showed that providing atypical group members once a stereotype is formed leads to this information being largely ignored, but if these group members are given whilst the stereotype is being formed it weakens the stereotype [93]. Thus, as it was the case in the current study, person knowledge is malleable by contradictory behaviour while it is still being formed, but less so when fully established.

As this is the first study investigating the activation of internal models of other people, some questions remain unanswered. First, the current study shows that, once established, internal models of other people’s behaviour are accessed fluently during action observation and bias the identification of the action towards these predictions. However, the range of situations and actors was by necessity restricted and the stimuli were relatively simplistic. It is therefore important to establish that people can also acquire internal models of others in real life social interactions, where participants meet a larger number of different individuals across a variety of more loosely connected situations that offer a variety of action possibilities that nevertheless suggest similar underlying traits (e.g., sporty and academic situations such as libraries, lecture halls, and fitness studios).

Such studies would also help solve the second question, namely whether the mechanisms underlying these predictions are uniquely social or whether they rely on domain general mechanisms. As noted, humans routinely acquire even complex relationships between stimuli, social and otherwise, and can use them to predict what comes next e.g., reaching from artificial grammar sequences [36]. Several theorists argue that the internal models one builds of other people are very similar, implying a continuity between the learning mechanisms for physical and social causality [94–96]. A question is therefore whether the current results can be accounted for by more general non-social mechanisms as well, which, for example, simply learn the contingencies between subsequent stimuli, whether they are social or non-social.

Although this was not the focus of this first study, several aspects of our research suggest a reliance on at the very least action-specific information. First, our prediction effects were restricted to predictions of object-directed actions, but not withdrawals. While this finding is very much in line with prior findings of a special status of goal directed actions during both action observation and prediction (see above, and [5] for a review), it is hard to account for by abstract stimulus based learning, which should apply to all stimulus types equally. Second, we found that similar prediction effects (again restricted to actions towards objects) were obtained when person information was explicitly given in Experiment 2 in a decidedly social format: which actions the two actors typically carry out with the objects. It is hard to see why participants would have effortfully translated these descriptions into non-social contingences prior to the experiment, especially as they had to evaluate these person descriptions after each block. Finally, while the effect was weak and could only be demonstrated when data was pooled across groups, the response time prediction effects were weakly related to subsequent person ratings: how much the two individuals interacted with and liked the objects. Again, from the viewpoint of mere stimulus learning, such relationships would not be predicted. It suggests that at the very least some of the learning during the response time task is drawn upon when making person judgements, suggesting a social or at least action based encoding of the stimuli.

Future studies will need to more directly test to what extent uniquely social mechanisms underlie these prediction effects. The type of experiment specified above would help solve these questions. For example, after participants are exposed to two individuals either acting in sporty or academic situations, one could test to what extent the acquired knowledge is action based such that any predictive speed up transfers to an equivalent action that achieves the same goal (e.g., making notes on a computer or on a notepad) or reflects attribution of higher level personality traits that generalises even to new situations (i.e., different sporty or academic situations). Such studies would open up the possibility of more closely linking research in social and experimental psychology to provide a common predictive person model framework for human social interactions.

## Conclusions

This study reveals that observers routinely access both explicit and implicit knowledge about which actions the observed actor typically carries out in the given situation, which allows them to rapidly identify these expected actions. These data provide evidence for a person-specific social anticipation system, which tracks the actions that others exhibit towards the environment and uses them to predict their forthcoming actions, in a situation-specific manner. Our results support recent models in which action identification emerges from an interaction of bottom-up cues and such top-down expectations derived from prior knowledge about others’ behaviour in different situations.

## Supporting Information

S1 File an excel file for the raw data. (XLSX)

S2 File a word document explaining the column headings in the raw data. (DOCX)
