# Supplementary material for: The Things You Do: Internal Models of Others’ Expected Behaviour Guide Action Observation
Source: PLoS One. 2016 Jul 19;11(7):e0158910. doi: 10.1371/journal.pone.0158910 (PMC4951130; doi:10.1371/journal.pone.0158910)
Supplement: S2 File — (DOCX) [file pone.0158910.s002.docx]

The raw data for the three experiments can be found within the “Raw data” excel file.

“M” in the stimuli name refers to the male actor and “F” refers to the female actor. “Sit” refers to neutral stimuli (before the actor either interacted or withdrew from the object) with the computer and “stand” refers to neutral stimuli with the ball.

**Experiment 1a and 1b computer task data**

- The counterbalance A or C refers to the male typically kicking the ball and the female typically typing and the counterbalance B or D refers to the female typically kicking the ball and the male typically typing.
- The column “Code” refers to the name of the neutral photo, then the name of the action photo then the response button (1 representing the up arrow key and 2 representing the down arrow key) then the RT (followed by the SOA timing either of 150 or 850 in Experiment 1a only).
- The column “Time” refers to the total running time of the experiment.
- The column “TTime” refers to the full time of the trial.
- The column “Stim typ” refers to whether participants responded correctly (“hit”), incorrectly (“incorrect”) or did not respond (“miss”)

**Experiment 1a and 1b questionnaire data**

- The counterbalance A refers to the male typically kicking the ball and the female typically typing and the counterbalance B refers to the female typically kicking the ball and the male typically typing.
- In the “Gender” column 1 refers to participants being female and 2 refers to being male
- In the “Handedness” column 1 refers to being right handed and 2 refers to being left handed

**Experiment 2**

- The column “Procedure[Block]” refers to the counterbalance with “Counter1” refers to the male typically kicking the ball and the female typically typing and “Counter2” refers to the female typically kicking the ball and the male typically typing.
- The column “FemaleScale.RESP” refers to how much the participants agree with the hypothesis for the female hypotheses.
- The column “FemaleScale.RT” refers to the time it takes to respond to the above scale.
- The column “MaleScale.RESP” refers to how much the participants agree with the hypothesis for the male hypotheses.
- The column “MaleScale.RT” refers to the time it takes to respond to the above scale.
- The column “Order” refers to which the type of block seen (equal actions, hypotheses match, hypotheses mismatch) – see “Running[SubTrial]” for which block is seen based on the counterbalance
- The column “Subtrial” refers to the trial number (this restarts for each new block)
- The column “ActionandHyp.ACC” refers to whether the response is correct (“1”) or incorrect (“0”)
- The column “ActionandHyp.RESP” refers to which button they responded for each trial
- The column “ActionandHyp.RT” refers to the response times for each trial
- The column “ActorCode” refers to which actor was seen (1= male, 2= female)
- The column “CorrectKey” refers to which was the correct response (1=turn away, 2= interact)
- The column “Facing” refers to which direction the actor faced (1=left, 2= right)
- The column “FrequentEvent” refers to how frequent the action was within the block (1=atypical, 2=typical)
- The column “Image1” refers to what neutral photo was presented
- The column “Image2” refers to what action photo was presented
- The column “ObjectCode” refers to which object was presented (1=football, 2=computer)
- The column “Running[Subtrial] refers to the frequency of the actions (“Female types” = the female mostly types and the male mostly kicks and “Male types” is the opposite. “Equal” refers to equal amounts of all actions for all actors)
- The column “Utilise” refers to whether they interact or turn away (0= turn away, 1= interact)

**Experiment 2 questionnaire data**

- In the “Gender” column “F” refers to being female and “M” refers to being male
- In the “Handedness” column “R” refers to being right handed and “L” refers to being left handed
- Q1-21 are the answers for the social intelligence scale

For all three experiments the new variable Action Typicality was created which assigned trials into those that were Typical or atypical for the actor based on which counterbalanced condition they were in. Another new variable was created for Action type which assigned trials into Acting towards and withdrawal actions.

For the first two experiments the questionnaires referring to liking and frequency were computed into whether they were the objects that were mostly interacted with or mostly turned away from.
